# Correlation of leptin, neuropeptide Y and amylin in childhood obesity

**DOI:** 10.1186/1687-9856-2013-S1-P97

**Published:** 2013-10-03

**Authors:** Eun Young Kim, Sei Young Kim, Min Seon Choi, Kyung Hee Yi

**Affiliations:** 1 Department of Pediatrics, Chosun University School of Medicine, Gwangju, Korea; 2 Department of Pediatrics, Daejin Medical Center, Seongnam, Korea; 3 Department of Pediatrics, Wonkwang University Sabon Medical Center, Gunpo, Korea

**Keywords:** Leptin, Neuropeptide Y, Amylin, Childhood obesity

## Purpose

To investigate the correlation of the serum leptin, NPY, amylin in the childhood obesity.

## Methods

The body mass index (BMI), waist and hip circumference, blood pressure (systolic/diastolic), lipid profile, fasting glucose, insulin, leptin, neuropeptide Y(NPY), amylin were measured in 56 children (24 obese children and 32 non-obese controls). We calculated HOMA-IR and evaluated the relationship between each anthropometric data, metabolic bio-marker and diet regulating factor (leptin, NPY, amylin).

## Results

Insulin and total cholesterol, triglyceride, LDL-cholesterol levels of the obese group were significantly higher than those of the non-obese group (p<0.05). leptin, NPY, leptin/NPY ratio, amylin levels of the obese group were significantly higher than those of the non-obese group (p<0.05). leptin showed significant correlation with BMI (r= 0.379, p= 0.043), NPY (r= 0.377, p= 0.044), L/N ratio (r= 0.754, p= 0.000) in the obese group. amylin showed significant correlation with insulin (r= 0.400, p= 0.048), HOMA-IR (r= 0.459, p= 0.028) in the obese group.

## Conclusion

There is abnormality in central diet regulatory system caused by leptin and neuropeptide Y resistance in the obese group. And amylin showed significant correlation with insulin resistance

